# Prediction of mortality based on the EuroSCORE II model in patients undergoing cardiovascular surgery

**DOI:** 10.34172/jcvtr.025.33353

**Published:** 2025-12-17

**Authors:** Mahdi Daliri, Ziae Totonchi, Mahmood Sheikh Fathollahi, Shiva Khaleghparast, Samaneh Karimian

**Affiliations:** ^1^Heart Valve Disease Center, Rajaie Cardiovascular Institute, Tehran, Iran; ^2^Cardiovascular Research Center, Rajaie Cardiovascular Institute, Tehran, Iran; ^3^National Center for Health Insurance Research, Tehran, Iran; ^4^Cardiovascular Nursing Research Center, Rajaie Cardiovascular Institute, Tehran, Iran

**Keywords:** EuroSCORE II, Mortality, Cardiovascular surgery, Complications

## Abstract

**Introduction:**

This study aims to evaluate the performance of the EuroSCORE II (European System for Cardiac Operative Risk Evaluation) model in predicting mortality and postoperative complications in adult patients undergoing cardiac surgery.

**Methods:**

In this prospective longitudinal study, 1,173 patients who underwent cardiac surgery between August 2021 and May 2022 were included to assess the predictive accuracy of the EuroSCORE II model for mortality and 11 major cardiovascular complications. Patients were followed up for 30-day and in-hospital mortality, as well as for the occurrence of 11 major cardiovascular complications. The model’s discriminative power was evaluated using the area under the receiver operating characteristic (AUC-ROC) curve, while its calibration was assessed through the goodness-of-fit test (Hosmer–Lemeshow test).

**Results:**

The data analysis showed that the area under the ROC curve of the EuroSCORE II model, used to predict the post-cardiac surgery outcomes was>0.7 in 10 out of 12 outcomes, which indicates good discrimination power. The area under curve (AUC) for predicting mortality was 0.749. The model calibration was assessed through the Hosmer–Lemeshow (H-L) goodness-of-fit test. Other findings including sensitivity, specificity and cutoff were also calculated, revealing the fitness of the prediction model.

**Conclusion:**

According to the findings, considering the power of differentiation and calibration of the EuroSCORE II model in the studied population, this model remains a valuable risk stratification tool, integrating additional predictive models or clinical parameters may enhance accuracy for certain postoperative outcomes.

## Introduction

 Cardiovascular diseases (CVDs) are the leading cause of mortality worldwide, and cardiac surgery is frequently performed around the world. Like other types of surgery, cardiac surgery has multiple postoperative complications.^1^ Risk stratification is a vital aspect of cardiac surgery across the globe. Various models have been developed to predict clinical outcomes, such as morbidity and mortality.^2^

 The European System for Cardiac Operative Risk Evaluation (EuroSCORE), which was initially established in 1999, aimed to enhance the process of patient selection and subsequently gained widespread acceptance. Nonetheless, with advancements in perioperative and postoperative management, the discriminative capability and calibration of EuroSCORE I experienced a decline. Consequently, a revised iteration, EuroSCORE II, which demonstrates superior performance compared to EuroSCORE I in terms of risk stratification, was introduced in 2011.^3^

 The EuroSCORE II is a crucial tool for assessing the risks associated with cardiac surgery during the intraoperative and postoperative periods.^4^ The EuroSCORE evaluation is a widely used, practical, and simple disease predictor.^5^ As predictive models are increasingly implemented within clinical practice, it becomes progressively essential to systematically evaluate their efficacy and revise them as necessary. The heightened attention towards registries, real-world data, and the paradigm of learning healthcare systems is likely to promote more regular or potentially continuous evaluation of concept drift.^6^

 To use risk assessment models after cardiac surgery, it is important to determine whether the current indicators are suitable for our population, and if they need to be adjusted.^7^

 The EuroSCORE II model comprises three parts to determine the risk of mortality post-cardiovascular surgery in adult patients:

1. Patient-related factors: age, gender, renal dysfunction based on creatinine clearance, extracardiac arteriopathy, preoperative movement disorder, history of previous cardiovascular surgery, chronic pulmonary disease, active endocarditis, critical preoperative state, and insulin-dependent diabetes mellitus.
2. Cardiac-related factors: the New York Heart Association (NYHA), left ventricular function (LVEF%), a recent myocardial infarction (MI) and increased pulmonary artery blood pressure.
3. Operation-related factors: the urgency of surgery, and the severity of interventions.^5^

In a study in Bangladesh with 1403 patients, EuroSCORE II was identified as the top predictor of cardiac surgery death and was also interrelated to longer ICU stays, use of inotropes, stroke, de novo dialysis, and low output syndrome. A high EuroSCORE II was associated with an increased risk of late mortality.^4^ According to a review of 621 Greek patients undergoing cardiac surgery, EuroSCORE II is a good predictor of in-hospital mortality.^8^ Based on a study conducted on Dutch patients who underwent cardiac surgery, the results showed that the EuroSCORE II model was better at predicting mortality than other models.^9^ Another study conducted to assess the performance of the EuroSCORE II model beyond European data found that EuroSCORE II outperformed the risk prediction model of the Society of Thoracic Surgeons.^10^ Wang, in China, found that EuroSCORE II outperformed Logistic Euroscore in predicting mortality and major postoperative complications.^11^ In 2023, Silverborn et al stated that the EuroSCORE II demonstrated an acceptable level of discriminative accuracy when utilized within a substantial cohort of patients undergoing coronary artery bypass grafting (CABG). Nevertheless, it significantly overestimated the mortality risk of this study population, particularly among younger individuals.^3^

 Based on a literature review, the EuroSCORE II evaluation model is widely recognized as an effective predictor of mortality following cardiac surgery worldwide. Nonetheless, in Iran the research results on using EuroSCORE II have been contradictory and limited. In 2013, Ghafari et al discovered that the EuroSCORE II model effectively predicted mortality in 1000 patients AUC for predicting mortality was 0.87.^12^ Despite this, in 2017 Atashi et al found that the same model had low discriminating power (AUC = 0.66) in predicting mortality and complications in 1337 patients.^13^

 In light of the discrepancies and insufficient research in Iran, it was essential to undertake a study to ascertain the performance of the EuroSCORE II model. Furthermore, in other studies conducted to evaluate the performance of EuroSCORE worldwide, the focus has primarily been on mortality and a limited number of complications. However, in this prospective study, we examined mortality and 11 major cardiovascular complications that may arise following cardiac surgery.

 To this end, this study aimed to predict mortality and 11 major cardiovascular surgery complications in adult patients undergoing surgery at a single center in Iran. If desirable results are achieved, this model is recommended to be adopted as a permanent assessment tool for predicting mortality, making decisions on risk-taking and performing surgical procedures.

## Materials and Methods

 This prospective longitudinal study assessed the predictive performance of the EuroSCORE II model regarding mortality and 11 post-cardiac surgery complications in adult patients (aged 18-95 years) who underwent cardiovascular surgery at Rajaie Cardiovascular Institute. Data collection for this study was conducted from August 23, 2021, to May 20, 2022, following approval from the Ethics Committee (IR.RHC.REC.1400.070) and after obtaining informed consent from the patients. The sampling method employed was sequential.

###  Inclusion Criteria

 The study included individuals aged 18 to 95 years who underwent cardiac surgery between August 23, 2021 and May 20, 2022. The procedures encompassed a range of cardiac surgeries, including isolated coronary artery bypass grafting (CABG), repair or replacement of one, two, or three heart valves, CABG combined with valve repair or replacement, structural defect repair, the Maze procedure, cardiac tumor resection, the Bentall procedure, the David procedure, CABG combined with structural defect repair, CABG combined with aortoplasty, and aortic valve replacement (AVR) with aortoplasty.

###  Exclusion Criteria

 Exclusion criteria encompassed surgeries not accounted for in the EuroSCORE II model, incomplete patient data that hindered comprehensive record access (particularly in emergency cases), and situations where patient follow-up was not feasible for various reasons.

 The research involved collecting comprehensive patient data across cardiac surgery’s pre-operative to calculate EuroSCORE II, intra-operative for death during the surgery, and post-operative phases for death and complications. Each patient had two dedicated data collection worksheets, one for the pertinent components required for EuroSCORE II calculation before surgery and the other for recording follow-up data related to postoperative mortality and major complications.

###  Data Collection

 The study involved collecting comprehensive patient data across the pre-operative, intra-operative, and post-operative phases. Pre-operative data were used to calculate EuroSCORE II, intra-operative data captured mortality during surgery, and post-operative data recorded mortality and complications. Each patient had two dedicated data collection worksheets: one for pre-surgical components required for EuroSCORE II calculation and another for recording follow-up data related to post-operative mortality and major complications.

 EuroSCORE II values were calculated based on data extracted from electronic medical records and through direct inquiries with patients, their families, physicians, and healthcare providers. The collected data, comprising 18 components, were entered into the EuroSCORE II website (https://www.euroscore.org/index.php?id=17) to determine the score during the pre-operative phase for candidates undergoing cardiac surgery.

 Following the initiation of surgery, the research team conducted rigorous patient follow-ups to monitor outcomes, including mortality and surgery-related complications. This process began in the operating room, intensive care unit (ICU) and continued in surgical wards and other inpatient departments. Patients discharged within 30 days post-surgery were contacted via telephone to assess mortality and potential complications. Those who remained hospitalized due to surgery-related complications were closely monitored until discharge or mortality, with all complications meticulously documented.

###  Study Population

 The research sample initially comprised 1191 patients. However, based on exclusion criteria such as the unavailability of the New York Heart Association (NYHA) Functional Classification, the Canadian Cardiovascular Association (CCA) Functional Class, or congenital heart surgery, 18 patients were excluded. Ultimately, 1173 patient records were analyzed.

 The EuroSCORE II questionnaire’s validity has been established in previous studies. For instance, Ghafari et al conducted research in Iran, where the area under the curve for mortality was 0.87^12^.

###  Definitions of Outcomes

 Surgical mortality is defined as any death occurring during the initial hospitalization following surgery (in-hospital mortality) or within 30 days postoperatively.^10^ Respiratory infections are diagnosed based on radiological findings and clinical symptoms.^5^ Acute respiratory distress syndrome (ARDS) is characterized by bilateral diffuse pulmonary infiltration and is defined by a PaO₂/FiO₂ ratio of ≤ 200 within 48 hours post-surgery. Dialysis-dependent acute renal failure is identified by the sudden necessity for dialysis or an elevation in serum creatinine levels above 2 mg/dL.^11^ A prolonged intensive care unit (ICU) stay following cardiac surgery is defined as ICU hospitalization lasting five or more days.^4^ Prolonged mechanical ventilation is classified as a duration of ≥ 24 hours.^14^ Stroke is diagnosed in patients based on confirmation through non-contrast computed tomography (CT) scanning within 72 hours of the suspected event.^5^ Dialysis-dependent renal failure is diagnosed in patients requiring renal replacement therapy post-cardiac surgery.^5^ The need for reoperation is defined as a return to the operating room within 24 hours post-surgery to manage hemorrhage or to drain a significant chest or pericardial hematoma.^15^ Mediastinitis is classified as a sternal wound infection, diagnosed based on positive microbial cultures, evidence of inflammation, and characteristic symptoms such as fever and chest pain, typically manifesting within 30 days post-cardiovascular surgery.^16^ prolonged length of stay after cardiovascular surgery is defined as hospitalization for 12 days or more.^17^ ICU readmission is determined if a patient requires re-hospitalization in the ICU within two weeks post-surgery^18^, whereas hospital readmission is defined as the necessity for re-hospitalization within 30 days postoperatively.^19^

###  Statistical Analysis 

 Data were analyzed using SPSS (version 24) for Windows (IBM SPSS Inc., Chicago, IL, USA) and STATA (version 14.1). Quantitative data were expressed as mean ± standard deviation (SD) or median (interquartile range), while qualitative data were reported as frequency (%) with a 95% confidence interval (CI). Model performance, in terms of predictive accuracy or discrimination power, was assessed using the area under the receiver operating characteristic (ROC) curve (AUC) with a 95% CI. Additionally, the cut-off point, sensitivity and specificity were calculated to predict mortality and complications. Results were presented as Odds ratios (OR) with a 95% CI, and the goodness-of-fit test *P*-value was computed to evaluate model calibration. The data analysis was conducted in three phases to examine the frequency distribution and descriptive indices of EuroSCORE II components.

 This study provides a comprehensive evaluation of the EuroSCORE II model in predicting post-cardiac surgery mortality and complications, contributing to the refinement of risk stratification and patient management in cardiovascular surgery.

## Results

**Figure 1:**
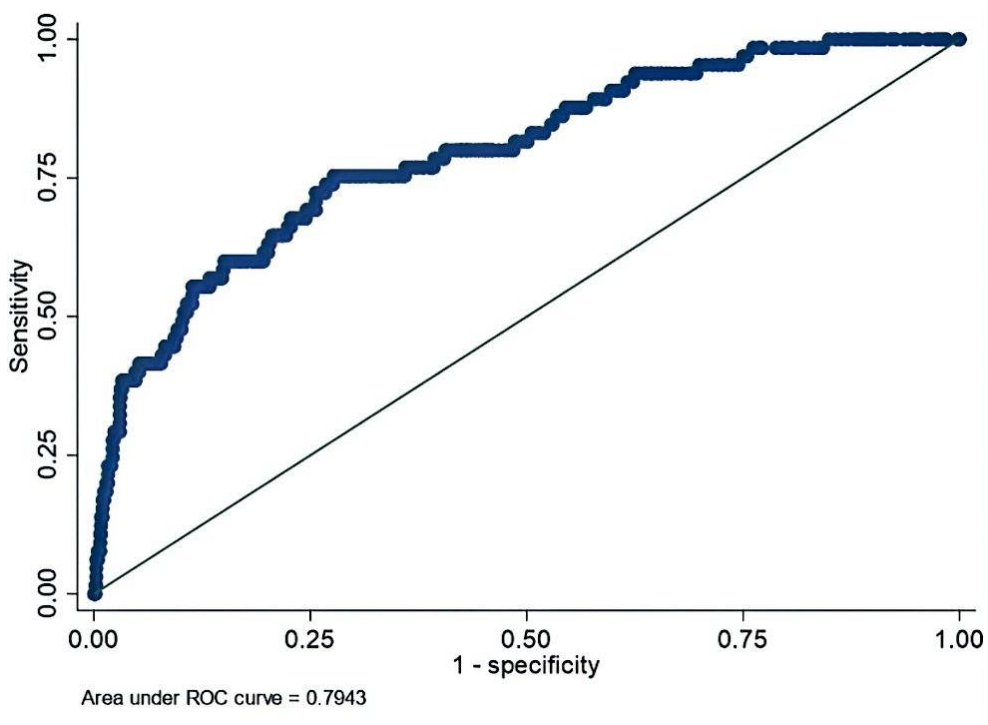
AUC-ROC curve for mortality occurring up to 30 days post-operation and in-hospital mortality diagnosis at varying EuroSCORE II model values (n = 1173)

 The demographic characteristics of 1173 patients and the results of the data analysis are shown in Tables 1–6 and the Figure 1.

**Table 1:** Frequency distribution and descriptive indices of EuroSCORE II components (n = 1173)

**EuroSCORE II components**		**Mean±Standard deviation,** **NO (%)**
Patient-related factors		
Age (Year)		57.10 ± 13.09 (ranged 18-92)
Weight (kg)		73.76 ± 3.81 (ranged 35-125)
Gender	Male	749 (63.9)
	Female	424 (36.1)
Chronic pulmonary disease		11 (0.9)
Extracardiac arteriopathy		45 (3.8)
Poor mobility		8 (0.7)
Previous cardiovascular surgery		102 (8.7)
Renal impairment	Normal: CC > 85ml/min	553 (47.1)
	Moderate impairment: 50ml/min < CC < 85ml/min	475 (40.5)
	Severe impairment: CC < 50ml/min	129 (11.0)
	Dialysis-dependent	16 (1.4)
Active endocarditis		16 (1.4)
Critical preoperative state		10 (0.9)
Diabetes on insulin		276 (23.5)
Cardiac-related factors		
NYHA Functional Classification	Ⅰ	128 (10.9)
	Ⅱ	629 (53.6)
	Ⅲ	346 (29.5)
	Ⅳ	70 (6.0)
Canadian Cardiovascular Association functional class Ⅳ		50 (4.3)
Left ventricular function (LVEF%)	Normal: EF > 50%	416 (35.5)
	Moderate: 31% < EF < 50%	628 (53.5)
	Poor: 21% < EF < 30%	101 (8.6)
	Very poor: EF ≤ 20	28 (2.4)
Recent myocardial infarction (MI)		159 (13.6)
Systolic Pulmonary arterial hypertension (PAH)	Normal	831 (70.8)
	Moderate: 31mmHg < PA < 55mmHg	283 (24.1)
	Severe: PA > 55mmHg	59 (5.0)
Surgery-related factors		
Urgency of surgery	Elective	984 (83.9)
	Urgent	64 (5.5)
	Emergency	125 (10.7)
	Salvage	0
Type of surgery	Coronary artery bypass surgery	686 (58.5)
	One valve repair or replacement	147 (12.5)
	Repair or replacement of More than one valve	105 (9.0)
	Coronary artery bypass surgery and valve(s) repair or replacement	102 (8.7)
	Aortoplasty	66 (5.6)
	Other	67 (5.7)
Thoracic aorta surgery		66 (5.6)

EuroSCORE II (European System for Cardiac Operative Risk Evaluation)- NYHA (New York Heart Association)- NO(Number).

**Table 2:** Frequency distribution of outcomes (n = 1173)

**Outcome**	**NO(%) Median (1**^st^** quartile-3**^rd^**quartile)**	**95% CI (%)**
Mortality (mortality occurring up to 30 days post-operation and in-hospital mortality)	65 (5.54)	4.23-6.85
Respiratory tract infection	87 (7.42)	5.92-8.92
Acute respiratory failure syndrome	12 (1.02)	0.45-1.6
Dialysis-dependent acute renal failure	30 (2.56)	1.65-3.46
Increasing ICU length of stay ( ≥ 5 days)	211 (17.99)	15.79-20.19
Mechanical ventilation duration (h) Range of variation	12 (10-16) 1-200	-
Cerebral complications	27 (2.31)	1.44-3.16
Need for re-operative	97 (8.27)	6.69-9.85
Sternal wound infection up to 30 days post-cardiovascular surgery	11 (0.94)	0.39-1.49
Prolonged length of stay after cardiovascular surgery ( ≥ 12 days)	323 (27.54)	24.98-30.09
Need for readmission to ICU within 2 weeks of the surgery	35 (2.98)	2.01-3.96
Need for readmission to hospital up to 30 days post cardiovascular surgery	11 (0.94)	0.39-1.49

CI (Confidence Interval)- ICU (Intensive Care Unit).

**Table 3:** Frequency distribution of mortality based on the surgery type (n = 65)

**Surgery type**	**NO (%)**
Coronary artery bypass surgery(CABG)	29 (4.2)
Repair or replacement of one valve	9 (6.1)
Repair or replacement of more than one valve	7 (6.7)
Coronary artery bypass surgery and valve(s) repair or replacement	10 (9.8)
Aortoplasty	8 (12.1)
Other	2 (3.0)

**Table 4:** AUC - ROC curve and the cut-off point for post-cardiovascular surgery outcomes (n = 1173)

**Outcome**	**AUC - ROC curve (95% CI)**	**Cut-off point**	**Sensitivity (%)**	**Specificity** **(%)**	**Classification accuracy (%)**	**Goodness-of-fit test-** **(** ***P*** **-value)**	**O/E ratio**
Mortality (mortality occurring up to 30 days post-operation and in-hospital mortality)	0.794 (0.736-0.853)	≥ 3.16	75.38	72.29	72.46	0.011	0.82
Respiratory tract infection	0.805 (0.758-0.853)	≥ 3.08	75.86	72.10	72.38	0.001	0.80
Acute respiratory failure syndrome	0.721 (0.574-0.867)	≥ 2.63	66.67	62.19	62.23	0.256	0.86
Dialysis-dependent acute renal failure	0.799 (0.715-0.882)	≥ 3.32	76.67	73.05	73.15	0.070	0.80
Increasing ICU length of stay ( ≥ 5 days)	0.718 (0.678-0.755)	≥ 2.61	65.88	67.36	67.09	0.001	0.94
Increased mechanical ventilation duration ( ≥ 24 hours)	0.804 (0.765-0.843)	≥ 3.05	73.08	73.15	73.15	< 0.001	0.86
Cerebral complications	0.788 (0.695-0.881)	≥ 3.45	77.78	74.17	74.25	0.036	0.80
Need for re-operative	0.715 (0.663-0.775)	≥ 2.86	67.01	68.31	68.20	0.032	0.90
Sternal wound infection up to 30 days post-cardiovascular surgery	0.737 (0.639-0.845)	≥ 2.87	72.73	66.01	66.07	0.363	0.95
Prolonged length of stay after cardiovascular surgery ( ≥ 12 days)	0.711 (0.678-0.744)	≥ 2.40	65.23	67.53	66.92	< 0.001	0.95
Need for readmission to ICU within 2 weeks of the surgery	0.596 (0.497-0.694)	≥ 2.33	60.00	57.73	57.80	0.257	0.99
Need for readmission to hospital up to 30 days post cardiovascular surgery	0.661 (0.550-0.771)	≥ 2.64	63.64	62.22	62.23	0.425	1.00

AUC - ROC curve (the area under the receiver operating characteristic (ROC) curve)- O/E ratio (Odds ratio)- Goodness of-fit-test (Hosmer-Lemeshow Test).

**Table 5:** Prediction of outcomes based on EuroSCORE II (n = 1173)

**Outcomes**	**OR** **(95% confidence interval)**	***P*** ** value**
Mortality (mortality occurring up to 30 days post-operation and in-hospital mortality)	1.126 (1.086-1.167)	< 0.001
Prolonged ICU length of stay ( ≥ 5 days)	1.131 (1.094-1.170)	< 0.001
Increased need for mechanical ventilation ( ≥ 24 hours)	1.173 (1.130-1.217)	< 0.001
Need for re-operative	1.097 (1.062-1.133)	< 0.001
Sternal wound infection	1.032 (0.973-1.093)	0.049
Respiratory tract infection	1.129 (1.091-1.169)	< 0.001
Acute respiratory failure syndrome	1.054 (1.015-1.095)	0.006
Dialysis-dependent acute renal failure	1.072 (1.036-1.110)	< 0.001
Cerebral complications	1.062 (1.028-1.097)	< 0.001
Need for readmission within 2 weeks of the surgery	1.024 (0.982-1.067)	0.264
Need for readmission to the hospital up to 30 days post-cardiovascular surgery	1.005 (0.907-1.114)	0.922
Prolonged length of stay after cardiovascular surgery ( ≥ 12 days)	1.114 (.078-1.151)	< 0.001

**Table 6:** The EuroSCORE risk stratifies patients.

**EuroSCORE II Risk stratifies**	**NO (%)**
Low risk = 0-2.99	795 (67.7)
Medium risk = 3-5.99	218 (18.7)
High risk = 6.0 and above	160 (13.6)

Table 1 illustrates the frequency distribution and descriptive indices of patient-related, cardiac-related, and surgery-related factors.

Table 2 shows the frequency distribution of outcomes. For instance, the first row shows that out of 1173 patients examined, 65 (5.54%) died. The other rows provide descriptive characteristics of each complication. The study found that the mean and standard deviation of EuroSCORE II in the population studied were 3.51 ± 5.26, ranging from 0.5 to 78.9.

Table 3 shows the frequency distribution of mortality based on the type of surgery. It displays that the highest percentage (12.1%) of death was related to aortoplasty surgery.

Table 4 presents the AUC-ROC curve and the cut-off point for post-cardiovascular surgery outcomes. The table includes values for sensitivity, specificity, accuracy of classification, goodness of fit test (*P*-value) for access model’s calibration, and the ratio of observed values to predicted values for each outcome, such as mortality and complications.

Figure 1 shows the AUC-ROC curve for mortality occurring up to 30 days post-operation and in-hospital mortality diagnosis at varying EuroSCORE II model values.

Table 5 presents the prediction of outcomes based on EuroSCORE II. As the EuroSCORE II increases, the likelihood of the result also increases. For instance, with a 1-point increase in the EuroSCORE II, the likelihood of the patient’s mortality becomes 1.126, meaning the chance of the patient’s mortality increases by 12.6%. Table 6 shows the EuroSCORE risk stratification in patients.

## Discussion

 The results indicated that in 10 out of 12 outcomes, the AUC-ROC curve exceeded 0.7, signifying that the model exhibited strong discrimination power. The EuroSCORE II model demonstrated a robust predictive ability for mortality and several major complications, including respiratory tract infection, acute respiratory failure syndrome, dialysis-dependent acute renal failure, prolonged ICU length of stay ( ≥ 5 days), increased mechanical ventilation duration ( ≥ 24 hours), cerebral complications, need for reoperation, sternal wound infection within 30 days post-surgery, and extended hospitalization ( ≥ 12 days) following cardiovascular surgery. These findings were supported by the area under the receiver operating characteristic (AUC-ROC) values.

 The model’s AUC for predicting mortality within 30 days post-surgery or during hospitalization was 0.794 (95% CI: 0.736–0.853), with a sensitivity of 75.38% and a specificity of 72.29%. These results suggest that EuroSCORE II possesses substantial discriminative power for identifying patients at risk of mortality, making it a reasonably effective tool for mortality prediction in this population. However, the observed-to-expected (O/E) ratio of 0.82 indicates a slight underestimation of mortality risk. The goodness-of-fit test results (*P*-value > 0.05) for complications such as acute respiratory failure syndrome, dialysis-dependent acute renal failure, sternal wound infection, need for ICU readmission, and hospital readmission suggest that the model is well-calibrated for these outcomes. However, the test results (*P*-value < 0.05) for mortality, respiratory infections, prolonged ICU stay, increased mechanical ventilation duration, cerebral complications, need for reoperation, and prolonged hospitalization indicate calibration issues. Despite the model’s strong discriminative ability for certain outcomes (as evidenced by AUC values), its calibration challenges suggest the need for adjustments or recalibration to enhance prediction accuracy. These findings highlight the necessity of refining the EuroSCORE II model, potentially by incorporating additional clinical variables or alternative risk stratification tools for specific postoperative complications.

 Notably, the low *P*-value of 0.01 for mortality in this model does not necessarily imply poor calibration. In the context of the Hosmer-Lemeshow test, an increase in sample size tends to reduce the p-value, making the test more sensitive to minor discrepancies between observed and predicted values. This sensitivity may lead to statistically significant results even when deviations are minimal, potentially resulting in the erroneous conclusion that the model is poorly calibrated when it remains practically useful.^20^

 Considering the values obtained for sensitivity (the accurate prediction of mortality or complications) and specificity at the EuroSCORE II cut-off point, the model effectively stratifies patients based on risk. Sensitivity represents the percentage of patients who died or experienced relevant complications with a EuroSCORE II value equal to or greater than the cut-off point. Conversely, specificity reflects the percentage of patients who survived or did not experience complications, with a EuroSCORE II value below the cut-off threshold. These findings confirm the model’s sufficient discriminatory power for the studied population, further supported by its sensitivity and specificity.

 Supporting evidence from international studies reinforces these conclusions. Research in Greece demonstrated that EuroSCORE II provides a highly accurate classification of patients, positioning it as a strong predictor of in-hospital mortality.^8^ Similarly, a study in the Netherlands reported superior performance of EuroSCORE II in most stratified analyses, confirming its strong discriminatory power for predicting mortality and complications.^9^ Our findings align with those of Ranjan et al affirming the model’s applicability for mortality, prolonged ICU stays, and dialysis dependence. However, they contrast with the model’s predictions for cerebral complications.^4^ Additionally, our results corroborate Wang et al’s study, which highlighted the model’s efficacy in predicting major postoperative complications, extended ICU stays, acute respiratory failure syndrome, and increased mechanical ventilation duration.^11^

 In the Iranian context, our results were consistent with those of Ghafari^12^, who confirmed the model’s accuracy in predicting mortality. However, they contradicted Atashi et al whose study reported suboptimal performance (AUC for mortality = 0.66, sensitivity = 61.88%, specificity = 66.23%).^13^ The alignment of our study’s AUC for mortality with global research underscores its reliability. Differences observed in complication predictions may be attributed to local population characteristics (e.g., genetic, sociocultural, and economic factors), variations in healthcare quality, and surgeon performance—factors not accounted for by the model. The discrepancy between our findings and Atashi et al’s^13^ study could stem from sample size, measurement errors, variations in surgical practices, errors in EuroSCORE II calculations, or patient follow-up challenges due to large sample sizes. Our study encountered limitations in fully addressing these influencing factors.

 To enhance the model’s applicability, it is recommended that EuroSCORE II be evaluated in larger cohorts over extended periods. Comparative studies incorporating alternative predictive models are also warranted. Furthermore, future research should investigate long-term mortality (e.g., six months to one year post-surgery) using these models. It is crucial to acknowledge that no predictive model can precisely forecast mortality following cardiac surgery. As demonstrated in this study, some patients with low EuroSCORE II values experienced complications or mortality, while others with high scores had uneventful recoveries. This variability underscores the necessity of further research incorporating newer models. With the introduction of EuroSCORE III, further validation and comparison with EuroSCORE II and other models remain imperative. Surgeons should employ EuroSCORE II as a supplementary tool alongside comprehensive patient evaluations when determining the optimal surgical approach.

 Based on the findings of this study, it is recommended that EuroSCORE II be utilized for all cardiac surgery patients due to its high discriminatory power, sensitivity, and specificity in predicting mortality and major postoperative complications. The model’s alignment with global research further supports its implementation. Preoperatively, EuroSCORE II should be integrated into clinical practice through a standardized assessment form completed by physicians or nurses, with the patient’s score documented in medical or electronic health records. This model can assist in surgical decision-making, patient selection, and risk assessment. However, given the inherent uncertainties associated with predictive models, as highlighted in this study and prior research, surgical decisions should always incorporate a holistic evaluation of multiple factors, including the clinician’s judgment of the patient’s overall condition.

## Conclusion

 Overall, the EuroSCORE II model appears to be a useful tool for predicting mortality and major complications, particularly respiratory infections, prolonged mechanical ventilation, and renal failure. However, its predictive performance is moderate for complications such as ARDS, prolonged ICU stay, and wound infections, and it is relatively weak in forecasting ICU readmission but it underestimated mortality risk. Therefore, while EuroSCORE II remains a valuable risk stratification tool, integrating additional predictive models or clinical parameters may enhance accuracy for certain postoperative outcomes.

## Competing Interests

 The authors have declared that they do not have any conflicts of interest.

## Ethical Approval

 This study was approved by the Research Ethics Committees of Rajaie Cardiovascular Medical and Research Institute (IR.RHC.REC.1400.070).
